# Scaling Up Evidence-Based Public Health Training

**DOI:** 10.5888/pcd15.180315

**Published:** 2018-11-21

**Authors:** Carol A. Brownson, Peg Allen, Samuel C. Yang, Kathryn Bass, Ross C. Brownson

**Affiliations:** 1 Prevention Research Center in St. Louis, Brown School, Washington University in St. Louis, St. Louis, Missouri; 2 National Association of Chronic Disease Directors, Decatur, Georgia; 3 Department of Surgery (Division of Public Health Sciences) and Alvin J. Siteman Cancer Center, Washington University School of Medicine, Washington University in St. Louis, St. Louis, Missouri

## Abstract

Evidence-based public health (EBPH) is the process of integrating science-based interventions with community preferences. Training in EBPH improves the knowledge and skills of public health practitioners. To reach a wider audience, we conducted scale-up efforts including a train-the-trainer version of the EBPH course to build states’ capacity to train additional staff. In this essay, we describe formats for course delivery and local adaptations to content, and we review success factors and barriers for state-based replication of the EBPH training course. Findings were based on our experiences and interviews. EBPH training was delivered in varied blended formats as well as in person and in distance courses, each with advantages and disadvantages. Adaptations were made to meet the needs of learners. Success factors included having committed and competent coordinators and trainers, organizational incentives, leadership support, funding, internal and external collaborators, the infrastructure to support training, and models to learn from. Barriers reported included insufficient staff or trainer capacity; time constraints for organizers, trainers, and participants; and lack of sustained funding. We hope our experience and findings will be a guide for states that are committed to building and sustaining capacity through continued EBPH training. Our lessons may also apply more generally to other workforce development training efforts.

## Introduction

Evidence-based public health (EBPH) has been defined as “the process of integrating science-based interventions with community preferences to improve the health of populations” (1). It involves many elements, including making decisions based on the latest scientific evidence on intervention effectiveness; using data and information systems systematically; applying program planning frameworks; engaging the community and a range of stakeholders in assessment and decision making; conducting quantitative and qualitative evaluation; and disseminating what is learned to decision makers (2–7). These skills are critical to effective practice and to meeting Public Health Competencies, Public Health Accreditation Board Standards for EBPH, and workforce development goals (8–10). To recognize and deliver EBPH, public health agencies need sufficient capacity (ie, the availability of resources and structures, and a prepared workforce) (5,11).

Training in EBPH improves the knowledge and skills of public health practitioners (5), and access to adequate training is a fundamental condition for making EBPH a reality in public health practice (12). Although there is a need for greater capacity in EBPH, training opportunities are often limited in number and reach. A focus on scaling up effective training programs (ie, expanding the reach to more people and places) is likely to 1) increase the impact of EBPH training (13,14); 2) result in a sufficient number (a “critical mass”) of practitioners trained to apply and sustain evidence-based practice, which in turn promotes the culture (values and beliefs) for EBPH practice (15–17); and 3) support effective approaches in EBPH training that can be adapted for different settings or populations (5).

Over the past 15 years, at least 20 evaluations of EBPH training courses have been published (5). Most of these programs are delivered in person, an effective yet resource-intensive method of training that has limited reach. In an effort to scale up training, some courses are offered in states in a train-the-trainer model (13,14), after which those states are expected to replicate the training for their coworkers and partners.

The advent of new learning technologies (eg, accessible video-conferencing platforms, podcasting, and social networking tools) creates new opportunities for distance learning and blended (digital plus instructor–student contact) formats. Demand also increases as technology-savvy workers face time and travel constraints. Blended learning opportunities have been increasingly applied in varied fields, including health and medicine (18–22), and it is reasonable to assume that trend will continue (23).

We describe alternative mechanisms (distance and blended formats) that states employed for replicating the EBPH training course, partners with whom they collaborated, local adaptations to the core content, and success factors and barriers to course replication. We seek to inform scale-up efforts of EBPH and other public health trainings.

## Gathering Information

We identified sites that had participated in train-the-trainer EBPH courses and had subsequently replicated the training. Sites included state health departments, state or regional public health training centers, and schools of public health. The sites were in 8 different states and reached state and local public health practitioners in 17 states. Invited site representatives included chronic disease practitioners, workforce development staff, school of public health faculty, and training center staff.

All sites had been provided technical assistance, and 6 of the 8 were part of a research project and received financial support for course replication. The other 2 sites had financial and in-kind support from other sources. Sites replicated the EBPH course at varying intervals from 2015 through 2017 and planned to continue offering courses in the future. Course intervals included biannual or annual delivery, several times a year, or open online availability. Evaluation included self-report pre–post surveys and key informant interviews.

In August and September of 2017, we conducted structured 30- to 60-minute telephone interviews with 13 training coordinators from 8 states. We identified state health department staff and university staff and faculty who were, or had been, responsible for planning and ensuring delivery of the EBPH course replication. Two people were interviewed from several sites to learn perspectives of both past and current course organizers or to learn perspectives from partnering course cosponsors. The structured interviews asked training coordinators to describe course delivery formats and experiences, facilitators and barriers to replication, advantages and disadvantages of distance learning versus face-to-face formats, and advice for others interested in providing EBPH trainings. Two team members coded each interview transcript, and the project team summarized coded texts and identified themes (18–20).

## What We Learned

### Alternative course formats

EBPH course replications by the 8 interviewed sites evolved from the original face-to-face course (typically 3.5 consecutive days) into various delivery formats. They were 1) distance on-demand course in one site, with all modules archived and available online; 2) blended distance course in one site with 9 archived online modules and weekly live web-based learning sessions over a 9-week period; 3) blended courses (distance combined with in-person) in 2 sites, with introductory content on select modules delivered remotely before the face-to-face session delivered in 2 to 2.5 consecutive days; and 4) fully face-to-face courses in 5 sites ranging from 2 to 5 days (Figure). The in-person courses were typically delivered on consecutive days, but one site held 3- to 4-day courses over 3 to 4 weeks, and another did 5 half days. One of the 8 sites delivered both blended and in-person courses in 2016 and 2017. Two sites that offered blended courses had also previously experimented with videoconferencing with participants in multiple physical locations over consecutive days.

**Figure:**
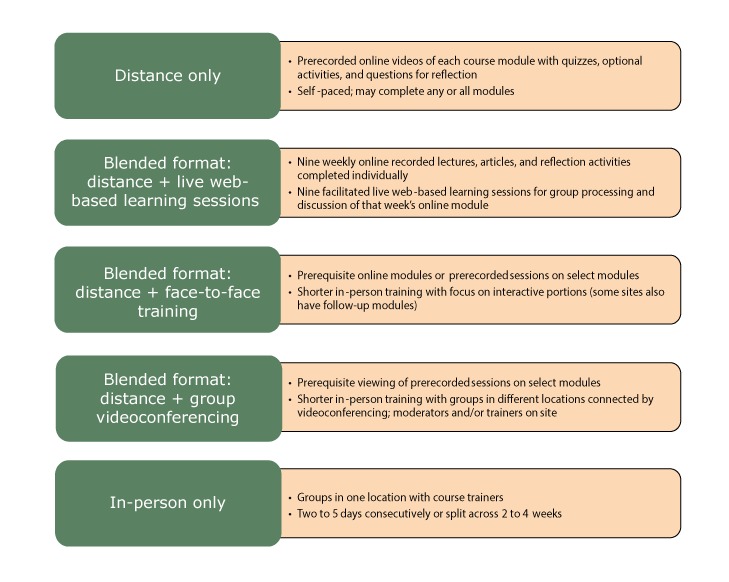
Evidence-based course alternative delivery formats.

### Pros and cons

Training coordinators reported advantages and disadvantages of different formats they tried. In-person courses offered opportunities to work with and learn from others, gave access to experts, and provided a forum for focused attention. Drawbacks included potential for information overload, time away from work, and travel costs. At the other end of the spectrum, distance-only courses had the reported advantages of further reach, self-paced learning (flexibility), cost savings, economy of scale, and having time between sessions to reflect and apply course content. Disadvantages mentioned included the loss of interaction with colleagues and working in isolation. The pros and cons of blended courses varied depending on the format; they shared some of the advantages and disadvantages of in-person and distance courses.

### Collaborators

States were encouraged to work with partners to replicate the course. The main collaborations EBPH training coordinators described were those between state health departments and university-based public health training centers. In 2 of the 4 states in which state or regional public health training centers led or co-led EBPH course delivery, training coordinators described the collaboration as co-planning course delivery and co-modifying course content. Most viewed state health departments as the main promoters and marketers of the course, as well as the main funders of the follow-up work to integrate EBPH processes into state and local health department practice. In several sites, directors of local health departments communicated local public health priorities and relevant topics to EBPH course organizers, who then used the information to tailor module data, examples, and exercises. State schools of public health provided content expertise and trainers, and in one state the school was the lead agency for the courses. Area health education centers (AHECs) collaborated in 2 states. In one state, AHECs provided continuing education credits for several disciplines of course attendees. In a second state, AHECs marketed courses, registered participants, and identified venues. Collaborations between entities often took the form of one or multiple designated staff members who held positions in both organizations, served as go-betweens, or sat on a shared workgroup or planning committee. Multiple training coordinators emphasized the importance of having a “point person” who could communicate across organizational lines and coordinate collaborations.

### Course content adaptations

States were encouraged to tailor the course to align with state or local priorities and initiatives and with the needs of their audiences. The most common content adaptation reported was using state and local examples to illustrate key points throughout the course. Many trainers also used state or local data to develop locally applicable exercises for the small-group work. Three sites developed a single case study for use throughout the entire course. Many training coordinators mentioned modifications to individual modules, such as simplifying the module on economic evaluation and using different databases in the module on quantifying the issue. Some added worksheets for the hands-on exercises and tip sheets for application. One site supplemented the course with additional content on adapting evidence-based approaches and assessing program sustainability. Another site added a list of suggestions on how to apply EBPH processes in local public health practice settings.

### Success factors

A key line of inquiry in the interviews concerned ingredients for success. The Table groups the facilitators into themes and provides quotes from training coordinators. Critical success factors were identified across the life cycle of the training course, beginning with the initial training. Training coordinators talked about the importance of mentorship, flexible core materials that could be locally tailored, and financial support for start-up costs. As coordinators took on responsibility for course replication, success factors included having committed and competent coordinators and trainers, organizational incentives, leadership support, funding, internal and external collaborators, infrastructure to support training, and models from which to learn.

**Table:** Facilitators for Successful Course Replication

Theme	Description and Illustrative Quotes
**Training, mentoring, and support** *Training coordinators indicated that having a core curriculum to work from, an initial train-the-trainer training, seed funding, and continued contact with the training team were all facilitators.*	
Original course material	“The core materials are so rich and great that I feel like it's made it easy for us to be able to replicate it and to implement it on an ongoing basis throughout the region.”
Initial train-the-trainer training	“Getting started, I would say that the train-the-trainer approach with having [PRC-StL] come over and actually hold the course in our state, with us and for us, was probably what got us off the ground.”
Access to PRC training team for technical assistance	“[PRC-StL] was just so available if we had any questions, and that was a huge benefit.”
Freedom to adapt the course	“Having someone to hand you their curriculum and say, we've taken it, we've adapted it, here, now you use this for where you're from and make it what you need it to be, is almost unheard of.”
Start-up funding	“The seed funds for that was icing on the cake. Probably the seed funds is probably what assured that we were able to do it.”
**Committed course replication team** *Training coordinators emphasized the importance of having a good course replication team, which consisted of having staff capacity (sometimes in the form of new hires), a growing pool of experienced trainers, and a point person for course planning and delivery.*	
Staff capacity	“That was always a team effort, completely committed to working together on this. And that has absolutely continued.”
Trainer capacity	“I do think one of the key things for us in the future is just the number of individuals that have been trained that have the skills now to be able to offer it. If we were a new state that just had one training, it'd be a lot more difficult to replicate it for the first time, I feel, but since we've had a number of trainings already in our state I feel that the capacity around the trained individuals that can offer the course is very beneficial.” “I would say that having both folks who are actively in practice right now or maybe they're now in the academic setting but previously were at a state or local health department has been really helpful. That we've gotten good feedback from participants that they felt like they could relate to the people that they were asking the questions of, that they had a good sense of the on-the-ground experience, if you will.”
Point person	“I think the biggest thing for me . . . is having [Organizer] coordinate the logistics, someone right here in our office . . . it’s having a point person who is familiar with the landscape of both academia and practice and has good connectivity with both. That's been key.”
**Organizational-level facilitators** *Training coordinators discussed internal factors that facilitated course replication. This often involved connecting the course to statewide EBPH efforts and accreditation processes, partnering with other health department divisions, using data and assessments to demonstrate or anticipate demand, and having alumni that would spread awareness and demand for the training.*	
Demand	“We did a workforce assessment . . . and basic public heath knowledge, that came up as one of our top training needs . . . there was a proven need for that. Many people were hungry to learn more about it. . . . Having any data that you can provide to leadership to show that there's a need for it I think is key.” “Having the need for the training — and not just because it's general and good public health practice but because there's a requirement from the state [to work on evidence-based strategies] — has . . . sustained the market.”
Accreditation requirements	“So, being able to position the course as one of the avenues for training that would be considered part of the public health accreditation process was really helpful in further engaging local health departments across the state.”
Internal collaboration	“[The workforce development] group is made up of individuals from all of our divisions . . . so that's very helpful in . . . disseminating information and also for bringing information as needs and requests back to the table for the department to attend to. . . . They're used to doing trainings, and it's just a good way to build on those systems within departments instead of having that training be held by one particular division.”
Alumni support	“There was the interest of participants, people that wanted to take the course. They'd heard from colleagues that, ‘Oh, it's great, I learned so much’ and on and on. We had champions for the course in the state, in the state health department, but also beyond.”
Commitment to application	“So, a sustained commitment from the state, sustained commitment from the [training center], and now it's part of our language in [the state], evidence-based public health decision making. It's just part of our language and how we do our work.” “[We wrote] the evidence-based public health course [into the requests for applications] as one of the trainings that people would go through. So, there's that kind of commitment and then the weaving it into funding opportunities.”
**Leadership**	*Training coordinators emphasized the importance of leadership that would support the program, increase demand, and commit to its delivery.* “I also was very fortunate to have strong support from the health officer, the state health officer. . . . They've been able to see the value of that. . . . It really, really helps when [the Association of State and Territorial Health Officials is] also promoting that kind of activity for public health staff, because it gets their attention and then they've got staff within their own health agency coming in saying, we need to do this.”
**Funding**	*Training coordinators mentioned funding as both a facilitator and an ongoing challenge, noting that extra funds were useful for keeping costs to participants low and sometimes allowing for additional capacity building.* “We've incorporated this whole training approach into [a workforce development center] that has been funded out of [a school of public health]. . . . It's a funder who's funding both the training and also funding communities or health departments . . . to do the work. And that's the ideal model, where you have that linkage.”
**Partnership**	*Training coordinators identified partnerships as a facilitator in providing everything from logistical support to meeting space to trainers and participants. Partners included regional public health training centers, universities, other health departments, and other bureaus within the state health department.* “I think another factor that really enabled us to continue was the ongoing partnership with the university. . . . We actually put in place a contract. . . . We bought the time of an epidemiologist, a medical epidemiologist, to augment our work in the state health department.”
**Infrastructure**	*The course was easier to replicate for training centers that have established teams, expertise, technology platforms, and a long-term commitment to providing trainings; organizations with experience have increased capacity.* “Economy of scale is the big piece of it. . . . It’s probably easier for us [a public health training center] to offer this on an ongoing basis . . . being able to offer it multiple times [a year].”
**Model programs**	*Learning from others was identified as a means to improve course facilitation and be more sustainable in providing and replicating the course.* “I would first look at other models of how to do it as a way to use the limited resources that we all have.”

Abbreviations: PRC, prevention research center; PRC-StL, Prevention Research Center in St. Louis.

### Barriers

The primary barriers to course replication reported by training coordinators fell into 3 broad categories: insufficient staff or trainer capacity; time constraints for organizers, trainers, and participants; and lack of sustained funding. Issues mentioned less frequently included lack of leadership buy-in; technology challenges for online sessions; difficulty matching content to meet differing needs of participants; insufficient follow-up to ensure application; scheduling challenges; and unclear communication among partners.

Challenges related to capacity included staff and trainer turnover, lack of designated staff time for replication, and finding qualified and willing trainers. Coordinators reported that staff turnover resulted in a loss of internal capacity for evidence-based practice and loss of EBPH trainers. It also affected the organizational capacity for hosting courses. Without a designated point person or dedicated staff time for replication, organizing courses proved inefficient and unsustainable. Finding available trainers with needed expertise and public health experience was also a challenge for some sites. Issues included lack of availability, difficulty with scheduling, and lack of incentives. This challenge was further heightened when experienced trainers left their positions. Quality trainers were considered especially valuable because subject matter experts were not always synonymous with good trainers.

Closely related to capacity challenges were time constraints. For organizers at the state level, workload was an issue. Tasks for course replication range from recruiting, scheduling, and coordinating trainers to recruiting and enrolling participants to managing the logistics of course delivery. Planning and organizing EBPH courses was often a new responsibility; if not formally assigned to a person or work unit, the task of organizing courses fell to staff members with already full workloads. Trainers from the work unit also found themselves with the added task of preparing for and teaching course modules, often as an uncompensated responsibility. For participants with busy work schedules, getting away for training was reported as a challenge, especially if it involved travel or leaving an office understaffed. For trainers based in academia, training the public health workforce was sometimes outside their expected roles, resulting in a lack of incentive to spend the time and effort.

Lack of sufficient and stable funding for EBPH training also undermined course delivery. Without financial support, committed staff found it difficult to make time for an unfunded program and sometimes had to abandon EBPH training in favor of their funded projects. If funding was insufficient or uncertain, patching together enough to fully fund a course was often a task unto itself.

## Advice for Replicating and Sustaining EBPH Training

Six critical success factors for replicating and sustaining EBPH training emerged:

1. Garner leadership and practitioner support for ongoing EBPH training. Suggestions included “walking the talk,” linking content to public health essentials, and building future training into the budget.
2. Sustain a large pool of skilled trainers.
3. Tailor the course to meet the learning needs of the intended audience.
4. Dedicate personnel time for course coordination.
5. Provide support for EBPH integration.
6. Consider web-based learning formats, which have the advantage of subdividing content into smaller units.

Specific to sustaining EBPH training, coordinators added these suggestions: maintain strong partnerships between the state health department and public health training centers, have a large cadre of experienced trainers from both academic and practice settings, and seek funding to continue the course.

## Summary and Implications for Practice

We now know a great deal about the importance of EBPH (the “why”) and effective training approaches (the “what”), but we know much less about replication and scale-up (the “how”). This article adds practice-based evidence to what we know about scaling up and sustaining EBPH training. Many training coordinators reported that EBPH training also served as the foundation and catalyst for additional training and technical assistance, thereby further increasing workforce capacity.

We found that the EBPH course is amenable to different formats, all of which have advantages and disadvantages for both organizers and participants (2,24–27). Course organizers in our project reported moving to blended courses to address concerns about information overload, time away from work, and costs of training; their desire to reach broader audiences and people in remote locations led to development of distance courses.

The success factors and barriers identified in this project are also instructive; they point to a need in public health to expand our commitment to workforce development in tangible ways. Identifying and cultivating public health “change agents” (people who are perceived as experts yet share common characteristics and goals with trainees) to become trainers or facilitators is one approach (28). Developing a range of administrative evidence-based practices (ie, leadership, organizational climate and culture, partnerships, workforce development, and financial processes) will enhance the likelihood of application and sustainability of evidence-based practices by individuals (16). At the same time, we also need to address the longstanding challenges of insufficient funding for workforce development and insufficient staff or trainer capacity to support EBPH and other public health training (29).

Another challenge is the wide range of experience and educational backgrounds among public health workers (30,31). Further study is needed on tailoring training content and modalities for practitioners with varied skills and education (32). While we continue to expand delivery formats for EBPH training, we need continued evaluation to more fully understand how various formats affect participants’ learning and application of evidence-based practices and to determine whether the modified formats improve sustainability of the training.

Although sustaining EBPH training has challenges, coordinators we interviewed demonstrated that health departments and their partners have found ways to continue training their workforce. Our findings can guide other states that are committed to building and sustaining capacity for EBPH practice. Lessons learned may also apply more generally to other workforce development training efforts.

## References

[1] Kohatsu ND , Robinson JG , Torner JC . Evidence-based public health: an evolving concept. Am J Prev Med 2004;27(5):417–21. 15556743 10.1016/j.amepre.2004.07.019

[2] Douglas MR , Lowry JP , Morgan LA . Just-in-Time training of the evidence-based public health framework, Oklahoma, 2016–2017. J Public Health Manag Pract 2018;1. 10.1097/PHH.0000000000000773 29521852

[3] Glasziou P , Longbottom H . Evidence-based public health practice. Aust N Z J Public Health 1999;23(4):436–40. 10.1111/j.1467-842X.1999.tb01291.x 10462873

[4] Jenicek M . Epidemiology, evidenced-based medicine, and evidence-based public health. J Epidemiol 1997;7(4):187–97. 10.2188/jea.7.187 9465542

[5] Brownson RC , Fielding JE , Green LW . Building capacity for evidence-based public health: reconciling the pulls of practice and the push of research. Annu Rev Public Health 2018;39(1):27–53. 10.1146/annurev-publhealth-040617-014746 29166243 PMC5972383

[6] Brownson RC , Fielding JE , Maylahn CM . Evidence-based public health: a fundamental concept for public health practice. Annu Rev Public Health 2009;30(1):175–201. 10.1146/annurev.publhealth.031308.100134 19296775

[7] Brownson RC , Gurney JG , Land GH . Evidence-based decision making in public health. J Public Health Manag Pract 1999;5(5):86–97. 10.1097/00124784-199909000-00012 10558389

[8] Bender K , Benjamin G , Carden J , Fallon M , Gorenflo G , Hardy GE Jr , Final recommendations for a voluntary national accreditation program for state and local health departments: steering committee report. J Public Health Manag Pract 2007;13(4):342–8. 10.1097/01.PHH.0000278026.49196.40 17563621

[9] Public Health Accreditation Board. Public health accreditation board standards: an overview. Alexandria (VA): Public Health Accreditation Board; 2011|.

[10] Public Health Accreditation Board. Modified version of the core competencies for public health professionals; 2017. http://www.phf.org/resourcestools/Pages/Modified_Core_Competencies_for_Public_Health_Professionals.aspx. Accessed April 22, 2018.

[11] Hanusaik N , Sabiston CM , Kishchuk N , Maximova K , O’Loughlin J . Association between organizational capacity and involvement in chronic disease prevention programming among Canadian public health organizations. Health Educ Res 2015;30(2):206–22. 10.1093/her/cyu062 25361958 PMC4364054

[12] Allen P , Jacob RR , Lakshman M , Best LA , Bass K , Brownson RC . Lessons learned in promoting evidence-based public health: perspectives from managers in state public health departments. J Community Health 2018;43(5):856–63. 10.1007/s10900-018-0494-0 29500725 PMC6119481

[13] Jacobs JA , Duggan K , Erwin P , Smith C , Borawski E , Compton J , Capacity building for evidence-based decision making in local health departments: scaling up an effective training approach. Implement Sci 2014;9(1):124. 10.1186/s13012-014-0124-x 25253081 PMC4180843

[14] Yarber L , Brownson CA , Jacob RR , Baker EA , Jones E , Baumann C , Evaluating a train-the-trainer approach for improving capacity for evidence-based decision making in public health. BMC Health Serv Res 2015;15(1):547. 10.1186/s12913-015-1224-2 26652172 PMC4676893

[15] Aarons GA , Hurlburt M , Horwitz SM . Advancing a conceptual model of evidence-based practice implementation in public service sectors. Adm Policy Ment Health 2011;38(1):4–23. 10.1007/s10488-010-0327-7 21197565 PMC3025110

[16] Brownson RC , Allen P , Duggan K , Stamatakis KA , Erwin PC . Fostering more-effective public health by identifying administrative evidence-based practices: a review of the literature. Am J Prev Med 2012;43(3):309–19. 10.1016/j.amepre.2012.06.006 22898125 PMC3990249

[17] Klein K , Sorra J . The challenge of innovation implementation. Acad Manage Rev 1996;21(4):1055–80. 10.5465/amr.1996.9704071863

[18] Edwards G , Kitzmiller RR , Breckenridge-Sproat S . Innovative health information technology training: exploring blended learning. Comput Inform Nurs 2012;30(2):104–9. 10.1097/NCN.0b013e31822f7f7a 21915046

[19] Liu Q , Peng W , Zhang F , Hu R , Li Y , Yan W . The effectiveness of blended learning in health professions: systematic review and meta-analysis. J Med Internet Res 2016;18(1):e2. 10.2196/jmir.4807 26729058 PMC4717286

[20] Mehta N , Geissel K , Rhodes E , Salinas G . Comparative effectiveness in CME: evaluation of personalized and self-directed learning models. J Contin Educ Health Prof 2015;35(Suppl 1):S24–6. 10.1002/chp.21284 26115238

[21] Moore GS , Perlow A , Judge C , Koh H . Using blended learning in training the public health workforce in emergency preparedness. Public Health Rep 2006;121(2):217–21. 10.1177/003335490612100220 16528957 PMC1525268

[22] Lewin LO , Singh M , Bateman BL , Glover PB . Improving education in primary care: development of an online curriculum using the blended learning model. BMC Med Educ 2009 10;9:33. 10.1186/1472-6920-9-33 PMC2702356 19515243

[23] Stacey E , Gerbic P . Success factors for blended learning. Proceedings Ascilite Melbourne 2008. Melbourne, Australia; 2008. p. 964-8.

[24] Ballew P , Castro S , Claus J , Kittur N , Brennan L , Brownson RC . Developing web-based training for public health practitioners: what can we learn from a review of five disciplines? Health Educ Res 2013;28(2):276–87. 10.1093/her/cys098 22987862 PMC3594926

[25] Bell M , MacDougall K . Adapting online learning for Canada’s Northern public health workforce. Int J Circumpolar Health 2013;72(1):72. 10.3402/ijch.v72i0.21345 23971012 PMC3749850

[26] Cook DA , Levinson AJ , Garside S , Dupras DM , Erwin PJ , Montori VM . Internet-based learning in the health professions: a meta-analysis. JAMA 2008;300(10):1181–96. 10.1001/jama.300.10.1181 18780847

[27] Mainor AG , Decosimo K , Escoffrey C , Farris P , Shannon J , Winters-Stone K , Scaling up and tailoring the “Putting Public Health in Action” training curriculum. Health Promot Pract 2018;19(5):664–72. 29191082 10.1177/1524839917741486 PMC6396877

[28] Proctor EK . Leverage points for the implementation of evidence-based practice. Brief Treat Crisis Interv 2004;4(3):227–42. 10.1093/brief-treatment/mhh020

[29] Wiesner PJ . Four diseases of disarray in public health. Ann Epidemiol 1993;3(2):196–8. 10.1016/1047-2797(93)90137-S 8269076

[30] Gebbie KM , Turnock BJ . The public health workforce, 2006: new challenges. Health Aff (Millwood) 2006;25(4):923–33. 10.1377/hlthaff.25.4.923 16835170

[31] Hilliard TM , Boulton ML . Public health workforce research in review: a 25-year retrospective. Am J Prev Med 2012;42(5 Suppl 1):S17–28. 10.1016/j.amepre.2012.01.031 22502923

[32] Morshed AB , Ballew P , Elliott MB , Haire-Joshu D , Kreuter MW , Brownson RC . Evaluation of an online training for improving self-reported evidence-based decision-making skills in cancer control among public health professionals. Public Health 2017;152:28–35. 10.1016/j.puhe.2017.06.014 28732323 PMC5966825

